# Programmed Cell Death-1 Polymorphisms Decrease the Cancer Risk: A Meta-Analysis Involving Twelve Case-Control Studies

**DOI:** 10.1371/journal.pone.0152448

**Published:** 2016-03-31

**Authors:** Wenjing Dong, Mancheng Gong, Zhirong Shi, Jianjun Xiao, Junkai Zhang, Jiewen Peng

**Affiliations:** 1 Department of Oncology, Zhongshan Affiliated Hospital of Sun Yat-sen University, Zhongshan, Guangdong 528403, China; 2 Department of Urology, Zhongshan Affiliated Hospital of Sun Yat-sen University, Zhongshan, Guangdong 528403, China; 3 Department of Pharmacy, The Second People’s Hospital of Zhuhai, Zhuhai, Guangdong 519020, China; Duke Cancer Institute, UNITED STATES

## Abstract

Programmed cell death-1 (*PD-1*) plays an important inhibitory role in anti-tumor responses, so it is considered as a powerful candidate gene for individual’s genetic susceptibility to cancer. Recently, some epidemiological studies have evaluated the association between *PD-1* polymorphisms and cancer risk. However, the results of the studies are conflicting. Therefore, a meta-analysis was performed. We identified all studies reporting the relationship between *PD-1* polymorphisms and cancers by electronically searches. According to the inclusion criteria and the quality assessment of Newcastle-Ottawa Scale (NOS), only high quality studies were included. A total of twelve relevant studies involving 5,206 cases and 5,174 controls were recruited. For *PD-1*.*5* (rs2227981) polymorphism, significantly decreased cancer risks were obtained among overall population, Asians subgroup and population-based subgroup both in TT vs. CC and TT vs. CT+CC genetic models. In addition, a similar result was also found in T vs. C allele for overall population. However, there were no significant associations between either *PD-1*.*9* (rs2227982) or *PD-1* rs7421861 polymorphisms and cancer risks in all genetic models and alleles. For *PD-1*.*3* (rs11568821) polymorphism, we found different cancer susceptibilities between GA vs. GG and AA vs. AG+GG genetic models, and no associations between AA vs. GG, AA+AG vs. GG genetic models or A vs. G allele and cancer risks. In general, our results firstly indicated that *PD-1*.*5* (rs2227981) polymorphism is associated a strongly decreased risk of cancers. Additional epidemiological studies are needed to confirm our findings.

## Introduction

Programmed cell death-1 (*PD-1*), a member of the CD28/B7 superfamily of costimulatory molecules, is expressed on activated CD4+ and CD8+ T cells, natural killer T (NKT) cells, B cells, activated monocytes and some dendritic cell (DC) [1]. The human gene encoding *PD-1* is located on chromosome 2q37.3, which encodes a 50–55 kD type I transmembranous glycoprotein protein [2, 3]. The *PD-1* is consisted of an immunoglobulin-like extracellular domain, and a cytoplasmic domain containing an immunoreceptor tyrosine-based inhibitory motif (ITIM) and immunoreceptor tyrosine-based switch motif (ITSM) [4]. *PD-1* has been well characterized as a negative regulator of T cells, and when interacts with its two ligands PD-L1 (B7-H1) and PD-L2 (B7-DC), it can strongly inhibit both proliferation and cytokine production by CD4 and CD8 T lymphocytes [5, 6]. PD-L1 has been reported to be expressed on a variety of tumor tissues or cell lines, including breast cancer, cervical cancer, gastric carcinoma, esophageal cancer and laryngocarcinoma [7–11]. In addition, *PD-1* is importantly involved in the regulation of regulatory T-cells (Treg) function in cancer patients. Recently, some studies have revealed a direct relation between *PD-1* blockade and down-regulation of intracellular FoxP3 expression by Treg to correct immune escape in various types of tumors [12–14]. Based on the inhibitory role of *PD-1* in anti-tumor responses, we considered the *PD-1* gene (Gene bank ID: 5133) as a powerful candidate for genetic susceptibilities of individuals to cancers. Previous, most studies researched about the association between the *PD-1* polymorphisms and several autoimmune diseases, including type 1 diabetes (T1D), ankylosing spondylitis (AS), SLE and rheumatoid arthritis (RA) [15–18]. In recent years, some studies have been changed the focus on the role of *PD-1* polymorphisms in various types of cancer patients. To date, several single nucleotide polymorphisms (SNPs) have been reported for the *PD-1* susceptibility of cancers in literature, such as *PD-1*.*5* (rs2227981), *PD-1*.*9* (rs2227982), *PD-1* rs7421861 and *PD-1*.*3* (rs11568821) et al. However, the association between the *PD-1* polymorphisms and cancer risk is inconsistent. To clarify this issue, we performed a meta-analysis from all eligible studies, to assess the association of the *PD-1* polymorphism with cancer risk.

## Materials and Methods

### Primary search strategy and Inclusion Criteria

We identified all studies reporting the relationship between *PD-1* polymorphisms and cancers published before December 22, 2015 by electronically searches. The databases include Pubmed, EMBASE, the Cochrane Library database, Google Scholar, China National Knowledge Infrastructure (CNKI) and Wan Fang. The search strategies were based on combinations of the following key words: (‘‘Programmed death-1” or ‘‘*PD-1*”) and (‘‘cancer” or ‘‘carcinoma”) and (‘‘gene” or ‘‘allele” or ‘‘genotype” or ‘‘mutation” or ‘‘variant” or ‘‘variation” or ‘‘polymorphism”), without any restriction on language. The reference lists of reviews and retrieved articles were also searched by hand for additional articles. We did not enroll abstracts or unpublished studies. For inclusion, the studies must have met the following criteria: (1) studied on human beings; (2) clear objective in the relation between *PD-1* polymorphisms and cancer; (3) case-control study, regardless of sample size, using a hospital-based or a population-based design; (4) sufficient published data about the size of the sample, odds ratio (OR), and their 95% confidence intervals (CIs).

### Data Extraction

Data were carefully and independently extracted from all eligible publications by three of the authors (Wenjing Dong, Zhirong Shi and Jianjun Xiao). Any disagreement was resolved by discussion among the authors. All eligible data were listed in Table 1: the surname of the first author, date of publication, quality scores, ethnicity, sources of controls, number of cases and controls and the P value of Hardy-Weinberg Equilibrium (*HWE*). Different ethnicities were categorized as Asian and Caucasian. Study designs were stratified to population-based studies and hospital-based studies.

**Table 1:** Characteristics of eligible studies in the meta-analysis of *PD-1* polymorphisms and cancer risk.

Author	Year	Quality scores	Ethnicity	Cancer type	Design	Case total	CC	CT	TT	Control total	CC	CT	TT	*P* HWE
***PD-1*.*5* (rs2227981)**														
Ivansson EL	2010	6	Caucasians	cervical cancer	PB	1300	471	603	226	810	257	375	178	0.064
Haghshenas MR	2011	6	Asians	breast cancer	PB	435	194	191	50	328	137	145	46	0.446
Zhang H	2011	6	Asians	breast cancer	PB	486	295	169	22	478	244	210	24	0.012
Mojtahedi Z	2012a	6	Asians	colon cancer	PB	175	47	102	26	200	75	89	36	0.290
Mojtahedi Z	2012b	6	Asians	rectal cancer	PB	25	12	7	6	200	75	89	36	0.290
Savabkar S	2013	5	Asians	gastric cancer	HB	122	50	66	6	166	89	70	7	0.136
Yin L	2014	7	Asians	lung cancer	PB	324	198	106	20	330	181	105	44	0.000
Ma Y	2015	6	Asians	lung cancer	PB	528	244	216	68	600	256	246	98	0.004
***PD-1*.*9* (rs2227982)**						**Case total**	**CC**	**CT**	**TT**	**Control total**	**CC**	**CT**	**TT**	
Zhang H	2011	6	Asians	breast cancer	PB	487	111	249	127	506	95	268	143	0.121
Qiu H	2014	6	Asians	esophageal cancer	HB	616	159	303	154	681	189	325	167	0.245
Tang WF	2015	6	Asians	gastric cancer	HB	330	75	168	87	603	163	292	148	0.448
Ma Y	2015	6	Asians	lung cancer	PB	528	343	148	37	600	404	168	28	0.056
***PD-1* rs7421861**						**Case total**	**TT**	**CT**	**CC**	**Control total**	**TT**	**CT**	**CC**	
Zhang H	2011	6	Asians	breast cancer	PB	490	333	146	11	512	370	130	12	0.885
Qiu H	2014	6	Asians	esophageal cancer	HB	600	411	168	21	673	460	188	25	0.295
Tang WF	2015	6	Asians	gastric cancer	HB	324	226	91	7	598	408	168	22	0.368
Ge J	2015a	5	Asians	colon cancer	HB	199	133	60	6	620	440	163	17	0.685
Ge J	2015b	5	Asians	rectal cancer	HB	362	241	114	7	620	440	163	17	0.685
***PD-1*.*3* (rs11568821)**						**Case total**	**GG**	**GA**	**AA**	**Control total**	**GG**	**GA**	**AA**	
Haghshenas MR	2011	6	Asians	breast cancer	PB	436	365	63	8	290	231	55	4	0.726
Bayram S	2012	7	Asians	liver cancer	PB	236	191	45	0	236	180	56	0	0.039
Yousefi AR	2013	6	Asians	colon cancer	PB	80	18	27	35	110	43	45	22	0.114
Ma Y	2015	6	Asians	lung cancer	PB	528	426	102	0	600	456	142	2	0.009

PB, population-based controls; HB, hospital-based controls; *HWE*, Hardy–Weinberg equilibrium.

### Quality assessment

Three authors (Wenjing Dong, Zhirong Shi and Jianjun Xiao) assessed the study quality independently using the Newcastle-Ottawa Scale which is a star rating system [19]. Nine stars are defined as the full score, and 5 to 9 stars are usually considered to be a high methodological quality while 0 to 4 stars are considered to be a poor quality [20]. The quality of all enrolled studies was showed in Table 2. Any disagreements on the NOS score of the studies were resolved by discussion between the authors and our meta-analysis only enrolled high quality studies.

**Table 2:** Quality assessment based on the Newcastle-Ottawa Scale of studies included in this meta-analysis^a^.

Author	Year	Adequate definition of case	Representativeness of cases	Selection of control	Definition of control	Control for important factor or additional factor^b^	Exposure assessment	Same method of ascertainment for cases and controls	Nonresponse rate	Total quality scores
Ivansson EL	2010	★	★	★	★	★		★		6
Zhang H	2011	★	★	★	★	★		★		6
Haghshenas MR	2011	★	★	★	★	★		★		6
Mojtahedi Z	2012	★	★	★	★	★		★		6
Bayram S	2012	★	★	★	★	★★		★		7
Savabkar S	2013	★	★		★	★		★		5
Yousefi AR	2013	★	★	★	★	★		★		6
Yin L	2014	★	★	★	★	★★		★		7
Qiu H	2014	★	★		★	★★		★		6
Ma Y	2015	★	★	★	★	★		★		6
Tang WF	2015	★	★		★	★★		★		6
Ge J	2015	★	★		★	★		★		5

^a^A study can be awarded a maximum of one star for each numbered item except for the item Control for important factor or additional factor.

^b^A maximum of two stars can be awarded for Control for important factor or additional factor.

### Statistical Analysis

Crude ORs with corresponding 95% CIs were used to estimate the strength of the association between the *PD-1* polymorphisms and cancer risk. The significance of the pooled OR was determined by the *Z* test and *P* (two-tailed) <0.05 was considered statistically significant. Hardy-Weinberg equilibrium (*HWE*) in controls was calculated by chi-square test and *P*<0.05 signified a departure from *HWE*. Between-study heterogeneity was calculated by the *I*^*2*^ test. If the heterogeneity was statistically significant (*I*^*2*^>50%) [21], a random effect model (the DerSimonian and Laird method) [22] was used; otherwise, a fixed effect model (the Mantel-Haenszel method) [23] was applied. Subgroup analyses were performed by ethnicity and the control sources. The funnel plot and Egger’s test were both used to examine the publication bias. For the interpretation of Egger’s test, statistical significance was defined as *P*<0.05 [24].The statistical analysis was performed with STATA statistical software (Version 12.0; Stata Corporation, College Station, TX, USA).

## Results

### Study characteristics

**Fig 1:**
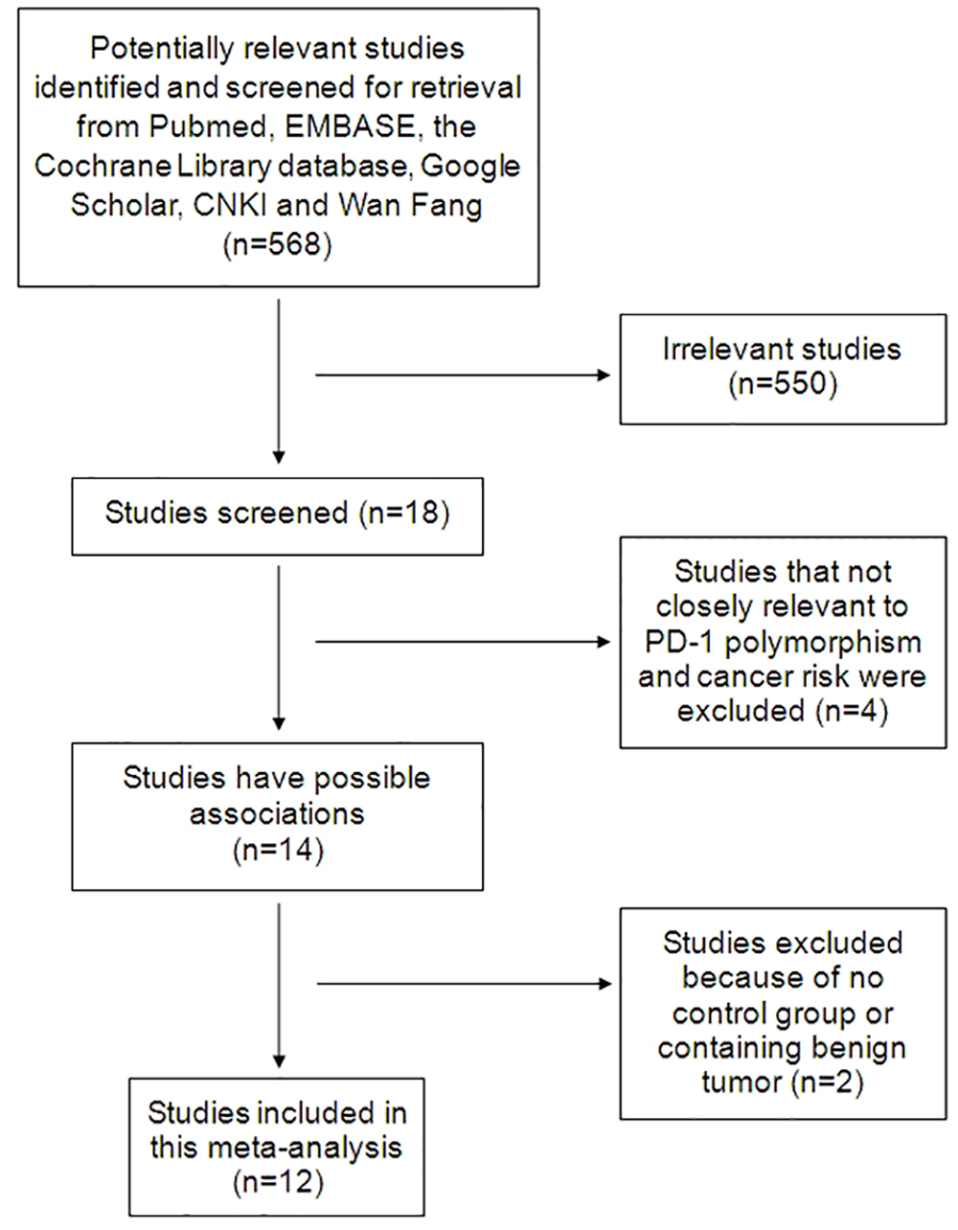
Flow diagram of study selection.

Five hundred and sixty-eight studies were retrieved after searching and screening based on our literature search strategy. There were 18 studies left when the irrelevant studies were excluded. Out of these, 14 studies had analyzed the association between the *PD-1* polymorphisms and cancers. After data extraction, one article [25] was excluded because of without control group while another one [26] was excluded as discussed about the gestational trophoblastic neoplasms, which contain both benign and malignant tumors. Hence we obtained 12 relevant studies that examined the association between the *PD-1* polymorphisms and cancer risk (Fig 1) [27–38]. All of them were evaluated by Newcastle-Ottawa Scale and met the high quality (Table 2). Overall, the meta-analysis included 5,206 cancer patients and 5,174 controls from 12 articles. The information extracted from all eligible articles was summarized in Table 1. All articles we included were case-control studies. Among them, breast cancer, gastric cancer, colorectal cancer and lung cancer are studied by two articles, respectively. The rest four studies are colon, esophageal, cervical and liver cancer study, respectively. Out of the 12 studies, 7 studies focused on the *PD-1*.*5* (rs2227981), while the *PD-1*.*9* (rs2227982), *PD-1* rs7421861 and *PD-1*.*3*(rs11568821) were all discussed in 4 studies, respectively. Among the 12 studies included in the meta-analysis, there were 11 studies of Asians and 1 study of Caucasians. According to the control source, only 4 were hospital-based researches, the rest 8 were population-based researches.

### *PD-1*.*5* (rs2227981)

**Fig 2:**
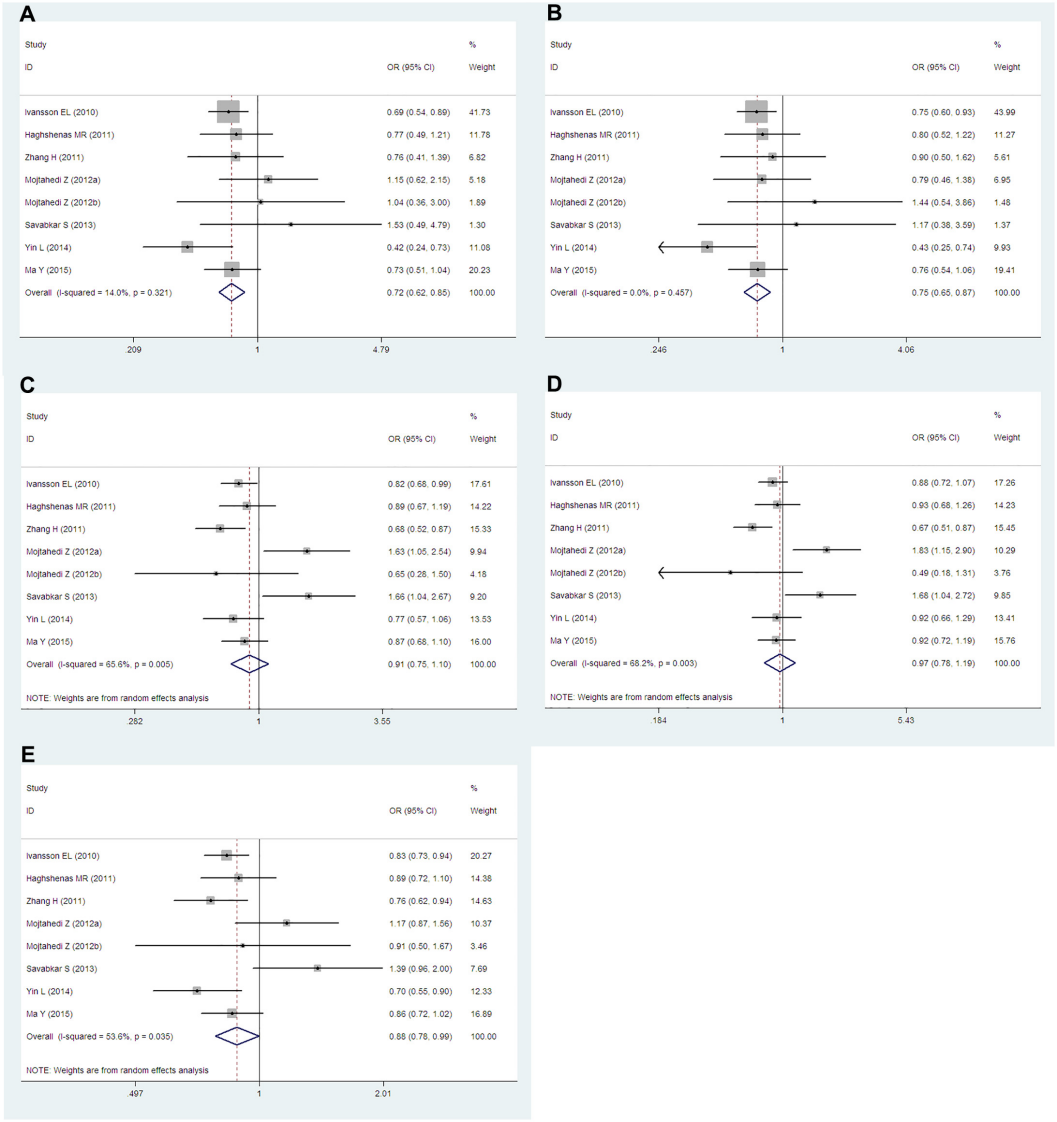
Forest plots of the *PD-1*.*5* (rs2227981) polymorphism and cancer risk for overall populations (A for TT vs. CC; B for TT vs. CT+CC; C for TT+CT vs. CC; D for TC vs. CC and E for T vs. C). The squares and horizontal lines correspond to the study-specific ORs and 95% CIs. The areas of the squares reflect the study-specific weights (which was the inverse of the variance). The diamonds represent the pooled ORs and 95% CIs.

**Fig 3:**
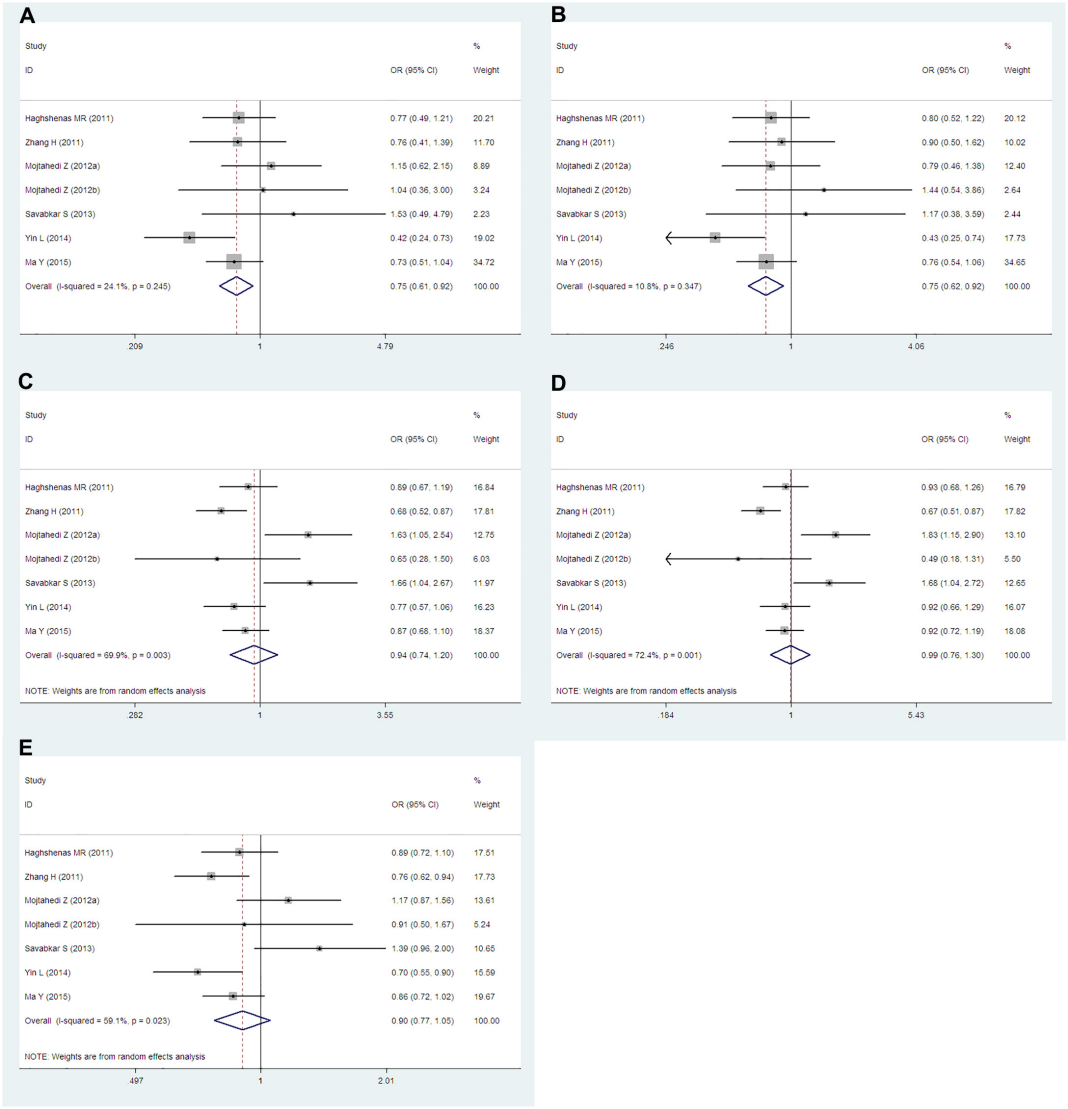
Forest plots of the *PD-1*.*5* (rs2227981) polymorphism and cancer risk for Asians subgroup (A for TT vs. CC; B for TT vs. CT+CC; C for TT+CT vs. CC; D for TC vs. CC and E for T vs. C). The squares and horizontal lines correspond to the study-specific ORs and 95% CIs. The areas of the squares reflect the study-specific weights (which was the inverse of the variance). The diamonds represent the pooled ORs and 95% CIs.

**Fig 4:**
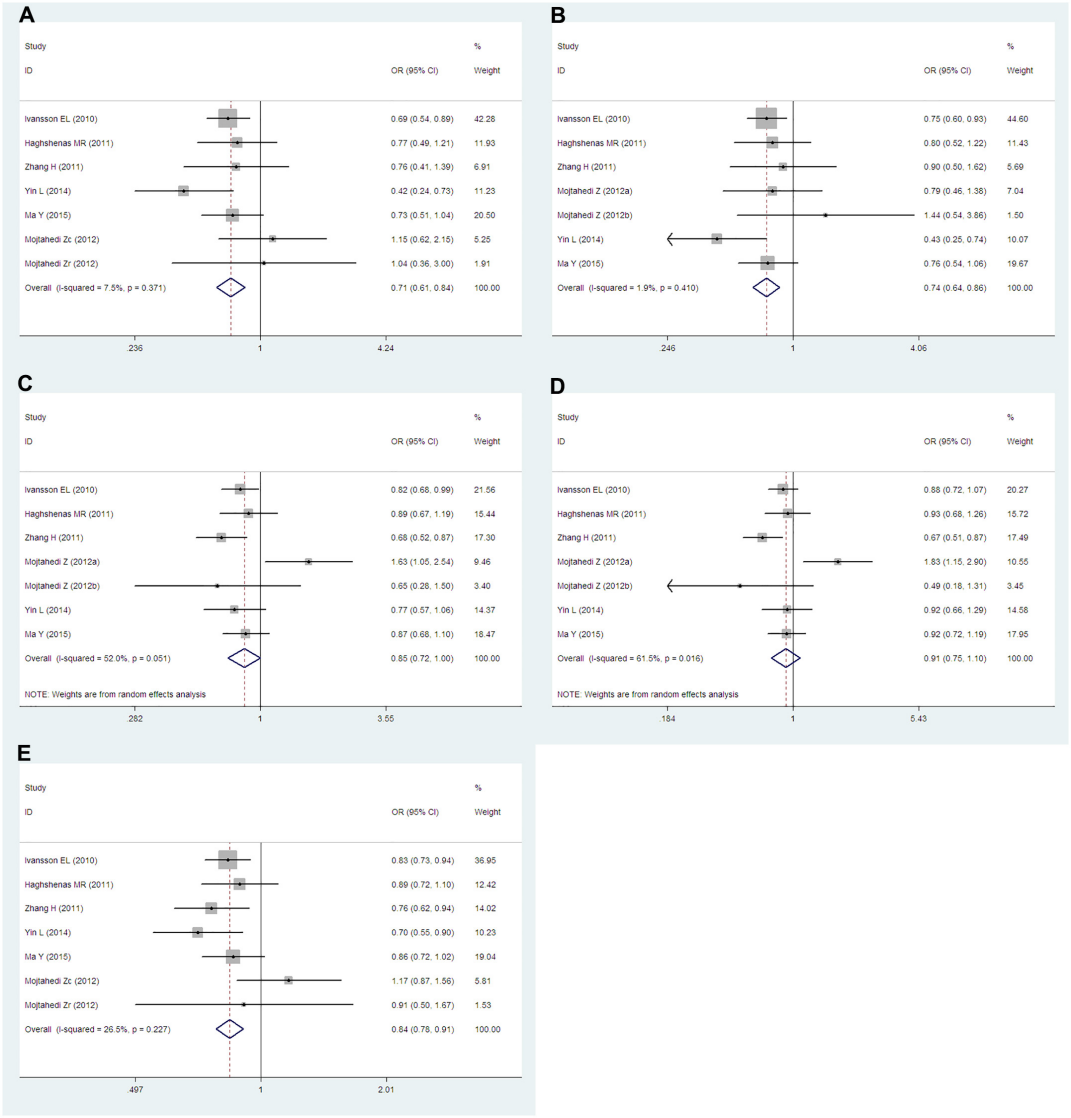
Forest plots of the *PD-1*.*5* (rs2227981) polymorphism and cancer risk for population-based subgroup (A for TT vs. CC; B for TT vs. CT+CC; C for TT+CT vs. CC; D for TC vs. CC and E for T vs. C). The squares and horizontal lines correspond to the study-specific ORs and 95% CIs. The areas of the squares reflect the study-specific weights (which was the inverse of the variance). The diamonds represent the pooled ORs and 95% CIs.

Data from seven studies which including 3,395 cases and 2,912 controls researched about the *PD-1*.*5* (rs2227981) were pooled together. Six of the studies were population-based and only one study was hospital-based. According to the ethnicity, six articles were researched about Asians and one study was about Caucasians. We conducted analyses for all genetic models and allele in overall group, Asians subgroup and population-based subgroup. Overall, we obtained significantly decreased cancer risks both in TT vs. CC (OR = 0.72, 95% CIs: 0.62–0.85, P = 0.000, *I*^*2*^ = 14.0%), TT vs. CT+CC (OR = 0.75, 95% CIs: 0.65–0.87, P = 0.000, *I*^*2*^ = 0.0%) genetic models and T vs. C (OR = 0.88, 95% CIs: 0.78–0.99, P = 0.04, *I*^*2*^ = 53.6%) allele. However, no dramatic associations were found in the other genetic models (TT+CT vs. CC: OR = 0.91, 95% CIs: 0.75–1.10, P = 0.343, *I*^*2*^ = 65.6%; TC vs. CC: OR = 0.97, 95% CIs: 0.78–1.19, P = 0.759, *I*^*2*^ = 68.2%) (Fig 2). When stratified by ethnicity, similar results were obtained in Asians subgroup. Cancer risks were remarkably reduced in TT vs. CC (OR = 0.75, 95% CIs: 0.61–0.92, P = 0.006, *I*^*2*^ = 24.1%) and TT vs. CT+CC (OR = 0.75, 95% CIs: 0.62–092, P = 0.005, *I*^*2*^ = 10.8%) genetic models. There were no significant associations in TT+CT vs. CC (OR = 0.94, 95% CIs: 0.74–1.20 P = 0.625, *I*^*2*^ = 69.9%), TC vs. CC (OR = 0.99, 95% CIs: 0.76–1.30, P = 0.959, *I*^*2*^ = 72.4%) and T vs. C (OR = 0.90, 95% CIs: 0.77–1.05, P = 0.190, *I*^*2*^ = 59.1%) (Fig 3). When considered the source of the control groups, we conducted analysis in population-based subgroup. Also, decreased cancer risks were found in TT vs. CC (OR = 0.71, 95% CIs: 0.61–0.84, P = 0.000, *I*^*2*^ = 7.5%), TT vs. CT+CC (OR = 0.74, 95% CIs: 0.64–0.86, P = 0.000, *I*^*2*^ = 1.9%) and T vs. C (OR = 0.84, 95% CIs: 0.78–0.91, P = 0.000, *I*^*2*^ = 26.5%). However, still we had observed no significant associations in TT+CT vs. CC (OR = 0.85, 95% CIs: 0.72–1.00, P = 0.054, *I*^*2*^ = 52.0%) and TC vs. CC (OR = 0.91, 95% CIs: 0.75–1.10, P = 0.335, *I*^*2*^ = 61.5%) (Fig 4).

### *PD-1*.*9* (rs2227982) and *PD-1* rs7421861

**Fig 5:**
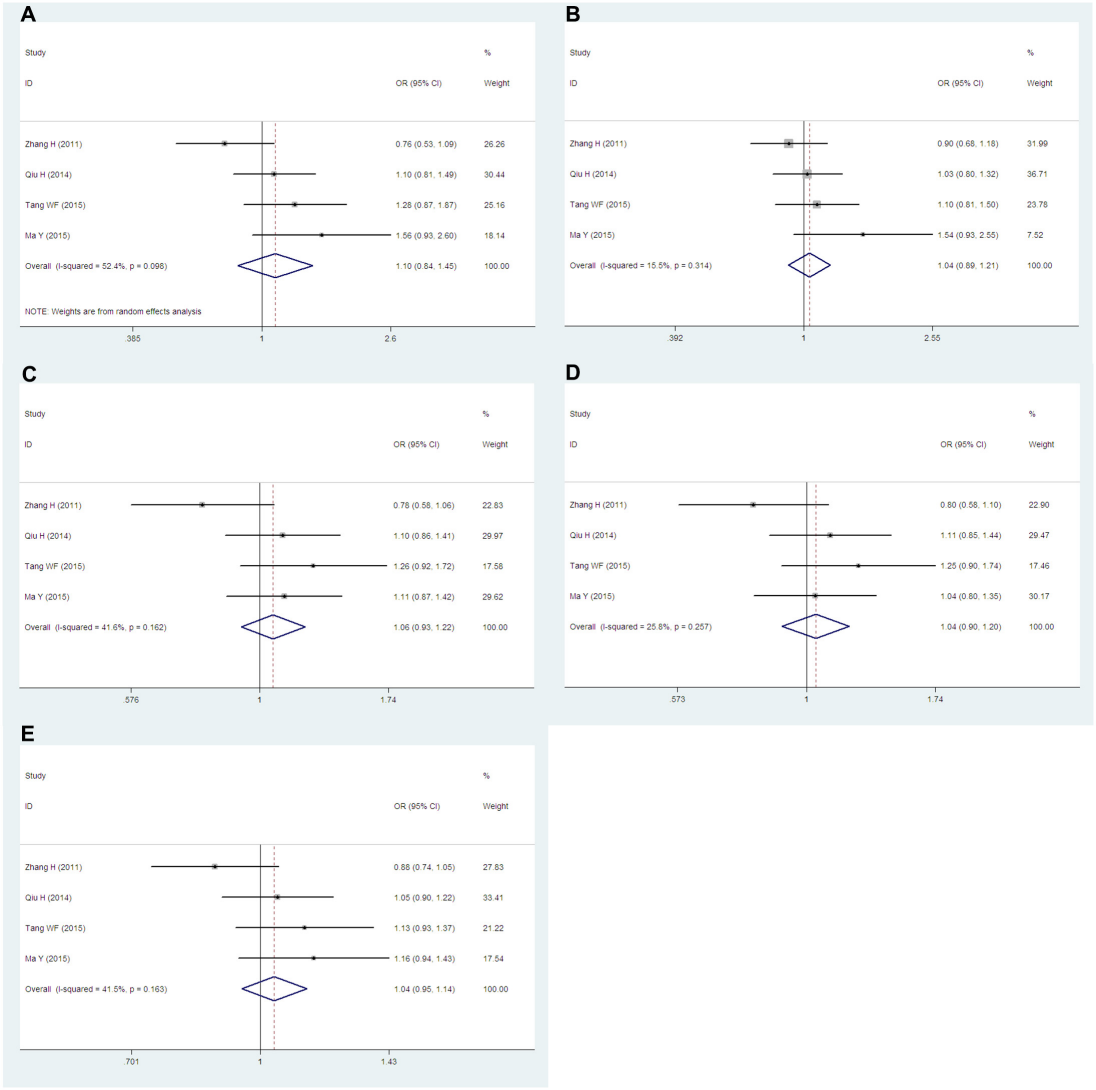
Forest plots of the *PD-1*.*9* (rs2227982) polymorphism and cancer risk for overall populations (A for TT vs. CC; B for TT vs. CT+CC; C for TT+CT vs. CC; D for TC vs. CC and E for T vs. C). The squares and horizontal lines correspond to the study-specific ORs and 95% CIs. The areas of the squares reflect the study-specific weights (which was the inverse of the variance). The diamonds represent the pooled ORs and 95% CIs.

**Fig 6:**
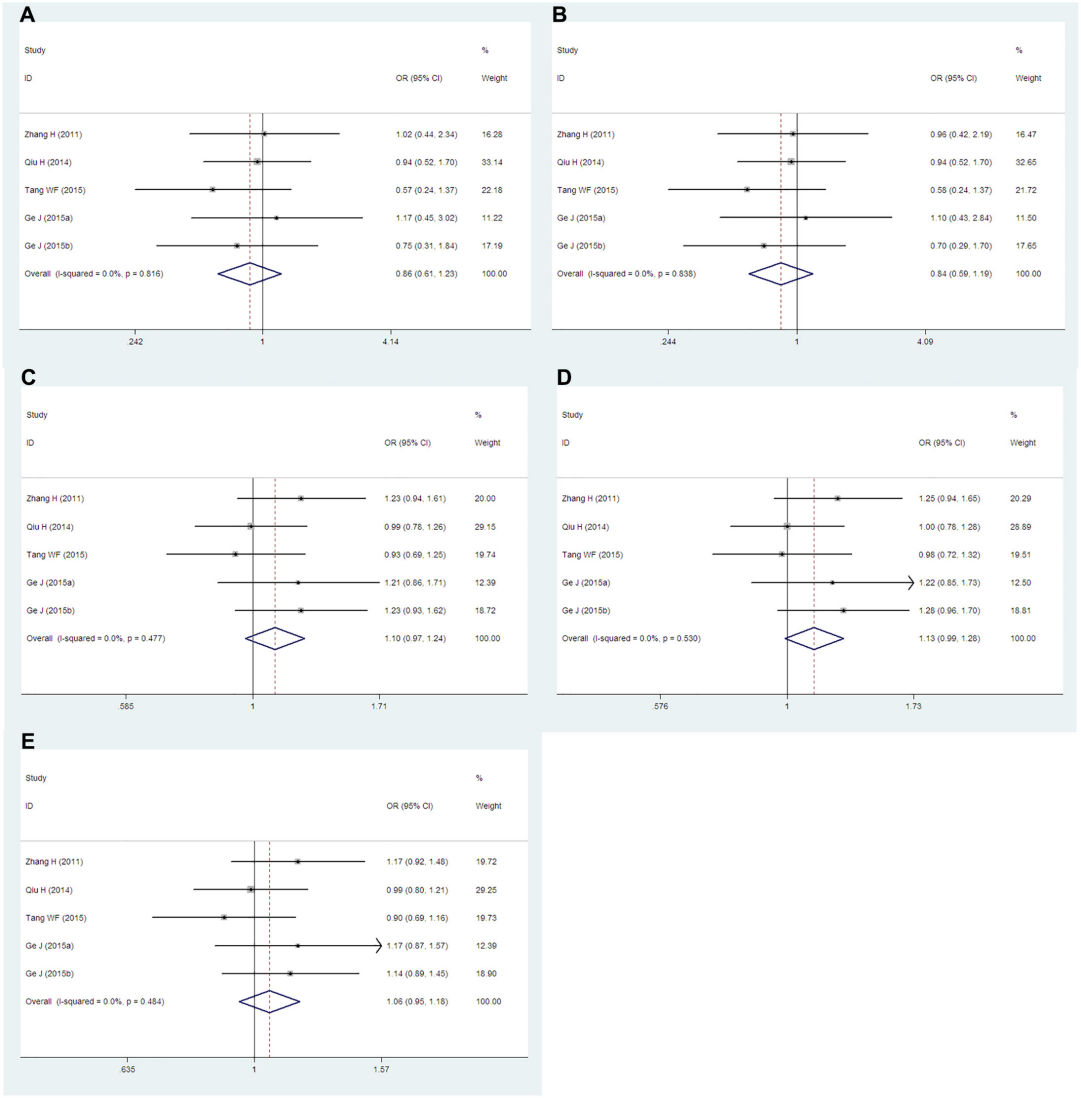
Forest plots of the *PD-1* rs7421861 polymorphism and cancer risk for overall populations (A for CC vs. TT; B for CC vs. CT+TT; C for CC+CT vs. TT; D for CT vs. TT and E for C vs. T). The squares and horizontal lines correspond to the study-specific ORs and 95% CIs. The areas of the squares reflect the study-specific weights (which was the inverse of the variance). The diamonds represent the pooled ORs and 95% CIs.

The *PD-1*.*9* (rs2227982) and *PD-1* rs7421861 polymorphisms were both discussed in four studies, which including 1,961 and 1,975 cases, and 2,390 and 2,403 controls, respectively. Overall, there were no significant associations between either *PD-1*.*9* (rs2227982) (Fig 5) or *PD-1* rs7421861 (Fig 6) and cancers in all genetic models and allele (*PD-1*.*9*: TT vs. CC: OR = 1.10, 95% CIs: 0.84–1.45, P = 0.487, *I*^*2*^ = 52.4%; TT vs. CT+CC: OR = 1.04, 95% CIs: 0.89–1.21, P = 0.609, *I*^*2*^ = 15.5%; TT+CT vs. CC: OR = 1.06, 95% CIs: 0.93–1.22, P = 0.399, *I*^*2*^ = 41.6%; TC vs. CC: OR = 1.04, 95% CIs: 0.90–1.20, P = 0.595, *I*^*2*^ = 25.8%; T vs. C: OR = 1.04, 95% CIs: 0.95–1.14, P = 0.393, *I*^*2*^ = 41.5%; *PD-1* rs7421861: CC vs. TT: OR = 0.86, 95% CIs: 0.61–1.23, P = 0.419, *I*^*2*^ = 0.0%; CC vs. CT+TT: OR = 0.84, 95% CIs: 0.59–1.19, P = 0.331, *I*^*2*^ = 0.0%; CC+CT vs. TT: OR = 1.10, 95% CIs: 0.97–1.24, P = 0.137, *I*^*2*^ = 0.0%; CT vs. TT: OR = 1.13, 95% CIs: 0.99–1.28, P = 0.072, *I*^*2*^ = 0.0%; C vs. T: OR = 1.06, 95% CIs: 0.95–1.18, P = 0.322, *I*^*2*^ = 0.0%). All the studies about these two polymorphisms are conducted in Asians. When concerning the control sources, there are two hospital-based and two population-based articles studied about the *PD-1*.*9* (rs2227982) polymorphism, while three hospital-based and one population-based article studied about the *PD-1* rs7421861 polymorphism.

### *PD-1*.*3* (rs11568821)

**Fig 7:**
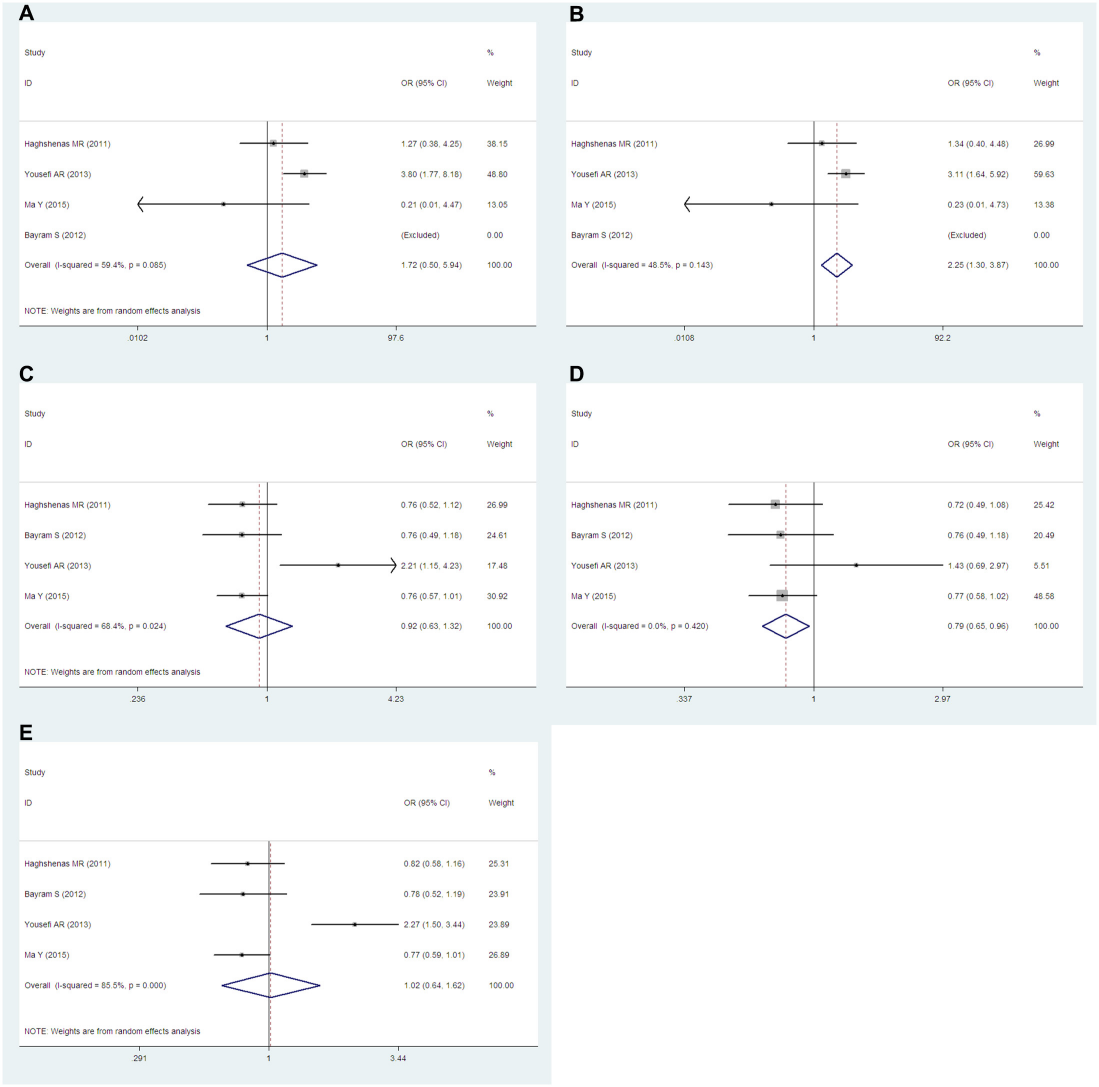
Forest plots of the *PD-1*.*3*(rs11568821) polymorphism and cancer risk for overall populations (A for AA vs. GG; B for AA vs. AG+GG; C for AA+AG vs. GG; D for AG vs. GG and E for A vs. G). The squares and horizontal lines correspond to the study-specific ORs and 95% CIs. The areas of the squares reflect the study-specific weights (which was the inverse of the variance). The diamonds represent the pooled ORs and 95% CIs.

There are four studies containing 1,280 cases and 1,236 controls discussed this polymorphism. All of these studies are population-based and conducted in Asians. Overall, a significantly decreased cancer risk was found in AG vs. GG genetic model (OR = 0.79, 95% CIs: 0.65–0.96, P = 0.021, *I*^*2*^ = 0.0%). Interestingly, an increased cancer risk was found in AA vs. AG+GG genetic model (OR = 2.25, 95% CIs: 1.30–3.87, P = 0.004, *I*^*2*^ = 48.5%). In addition, there were no associations between cancer risk and AA vs. GG (OR = 1.72, 95% CIs: 0.50–5.94, P = 0.394, *I*^*2*^ = 59.4%), AA+AG (OR = 0.92, 95% CIs: 0.63–1.32, P = 0.638, *I*^*2*^ = 68.4%) vs. GG or A vs. G (OR = 1.02, 95% CIs: 0.64–1.62, P = 0.945, *I*^*2*^ = 85.5%) (Fig 7).

### Publication bias

We performed both funnel plots and Egger’s tests for all genetic models and allele to assess the publication bias. Our results showed all the funnel plots were symmetrical distribution that suggested absence of publication bias (S1–S6 Figs). Also the results were supported by the Egger’s tests (S1 Table).

## Discussion

It is known to us that *PD-1* is an immune gene with potent inhibitory effects on immune cells. As an important gene for the “fine turning” of T lymphocyte activation and proliferation to affect host anti-tumor immunity, *PD-1* merits more investigations. Many studies have reported that over expression of *PD-1* is associated with poor prognosis in several tumors, which including breast, cervical, gastric, esophageal cancers and non-small cell lung cancer (NSCLC) [7–10, 39]. *PD-1* is expressed on tumor specific T cells, when interacts with PD-Ls, expressed on tumor and immune cells, could extensively restricts host anti-tumor immunity and creates antitumor suppressive milieu [40, 41]. Accordingly, it has been considered that blockade of *PD-1*-PDLs interaction as an immunotherapy procedure to conquer immune-suppression associated with cancer condition [41]. Recently, some studies had investigated the relationship between *PD-1* polymorphisms and various cancers including breast, gastric, colorectal, lung and liver cancer, et al. However, the results are controversial. So we performed this meta-analysis to discuss the associations between *PD-1* polymorphisms and cancer risk.

Previously, Mamat U et al. performed a meta-analysis [42] discussed the association between *PD-1*.*5* (rs2227981) polymorphism and cancer risks. Their results showed no association between *PD-1*.*5* (rs2227981) polymorphism and total cancer risk, but revealed an increased digestive system tumor risk. However, we found that they wrongly included one study researched about the *PD-1*.*3* (rs11568821) polymorphism and colon cancer risk [43] in their meta-analysis. Hence, it may significantly affect their total results and digestive system tumor subgroup results. In addition, they only enrolled six studies which including a wrong one and discussed the cancer risks with one polymorphism. By contrast, our meta-analysis included 12 relevant published studies and discussed the cancer risks with four polymorphisms. Moreover, our meta-analysis included higher numbers of the cases and controls than the prior one. In addition, we evaluated the quality of studies by Newcastle-Ottawa Scale and all the studies we included were met high quality, while the prior meta-analysis did not conduct any study quality assessment. So, our meta-analysis made a more convincing and detailed evaluation than the prior study did. All the characteristics and results of the present study for *PD-1*.*5* (rs2227981) polymorphism compared with the prior meta-analysis were summarized in Table 3.

**Table 3:** Characteristics and results of the present study compared with the previous meta-analysis.

		No. of studies		No. of cases		No. of controls		Overall results	
Polymorphism	Contrast	Previous	Present	Previous	Present	Previous	Present	Previous	Present
*PD-1*.*5* (rs2227981)	TT vs. CC	6	7	1,415	3,395	1,611	2,912	−	+
	TT vs. CT+CC							−	+
	TT+CT vs. CC							−	−
	TC vs. CC							−	−
	T vs. C							−	+
Asians Subgroup	TT vs. CC	NA	6	NA	2,095	NA	2,102	NA	+
	TT vs. CT+CC							NA	+
	TT+CT vs. CC							NA	−
	TC vs. CC							NA	−
	T vs. C							NA	−
Population-based Subgroup	TT vs. CC	NA	6	NA	3,273	NA	2,746	NA	+
	TT vs. CT+CC							NA	+
	TT+CT vs. CC							NA	−
	TC vs. CC							NA	−
	T vs. C							NA	−

+, positive result; −, negative result; NA, not available

In recent years, the application of the genome-wide association study (GWAS) in many types of diseases has exploded and lots of the GWASs about cancer risk were published. However, there is no GWAS focused on the *PD-1* polymorphisms and cancer risk. Therefore, our research mainly concerned on the case-control studies. In this study, association between *PD-1*.*5* (rs2227981), *PD-1*.*9* (rs2227982), *PD-1* rs7421861 or *PD-1*.*3* (rs11568821) and cancers risk were examined in all genetic models and allele, and all the results were summarized in Table 4. Concerning *PD-1*.*5*, our results showed a significant decreased cancer risks both in TT vs. CC and TT vs. CT+CC genetic models for overall population, Asians and population-based controls, also significant decreased cancer risk was found in T vs. C allele for overall population. *PD-1*.*5* located in exon 5, is a synonymous polymorphism that does not change final amino acid sequence of the protein. Thus these significant associations between *PD-1*.*5* and cancers probably may be *PD-1*.*5* variation linkage disequilibrium with other *PD-1* gene polymorphisms that may lead to alter the *PD-1* expression level [44]. Recently, Zhang Hua et al. [29] reported that the frequencies of CC genotype and C allele were higher in breast cancer patients than those in control individuals in Chinese population, and CC genotype and C allele may play a potential risk role in breast cancer. Consistently, our results indicated that in *PD-1*.*5*, TT genotype may reduce the cancers risk.

**Table 4:** Summary of meta-analyses of *PD-1* polymorphisms and cancer risk.

Group	Contrast	No. of studies	No. of cases	No. of controls	OR (95% CI)	Statistical method	*I*^*2*^%	*P*-value
***PD-1*.*5* (rs2227981)**								
Overall	TT vs. CC	7	3,395	2,912	0.72(0.62–0.85)	Fixed	14.0	0.000
	TT vs. CT+CC				0.75(0.65–0.87)	Fixed	0.0	0.000
	TT+CT vs. CC				0.91(0.75–1.10)	Random	65.6	0.343
	TC vs. CC				0.97(0.78–1.19)	Random	68.2	0.759
	T vs. C				0.88(0.78–0.99)	Random	53.6	0.040
Asians Subgroup	TT vs. CC	6	2,095	2,102	0.75(0.61–0.92)	Fixed	24.1	0.006
	TT vs. CT+CC				0.75(0.62–0.92)	Fixed	10.8	0.005
	TT+CT vs. CC				0.94(0.74–1.20)	Random	69.9	0.625
	TC vs. CC				0.99(0.76–1.30)	Random	72.4	0.959
	T vs. C				0.90(0.77–1.05)	Random	59.1	0.190
Population-based Subgroup	TT vs. CC	6	3,273	2,746	0.71(0.61–0.84)	Fixed	7.50	0.000
	TT vs. CT+CC				0.74(0.64–0.86)	Fixed	1.90	0.000
	TT+CT vs. CC				0.85(0.72–1.00)	Random	52.0	0.054
	TC vs. CC				0.91(0.75–1.10)	Random	61.5	0.335
	T vs. C				0.84(0.78–0.91)	Fixed	26.5	0.000
***PD-1*.*9* (rs2227982)**	TT vs. CC	4	1,961	2,390	1.10(0.84–1.45)	Random	52.4	0.487
Overall	TT vs. CT+CC				1.04(0.89–1.21)	Fixed	15.5	0.609
	TT+CT vs. CC				1.06(0.93–1.22)	Fixed	41.6	0.399
	TC vs. CC				1.04(0.90–1.20)	Fixed	25.8	0.595
	T vs. C				1.04(0.95–1.14)	Fixed	41.5	0.393
***PD-1* rs7421861**	CC vs. TT	4	1,975	2,403	0.86(0.61–1.23)	Fixed	0.0	0.419
Overall	CC vs. CT+TT				0.84(0.59–1.19)	Fixed	0.0	0.331
	CC+CT vs. TT				1.10(0.97–1.24)	Fixed	0.0	0.137
	CT vs. TT				1.13(0.99–1.28)	Fixed	0.0	0.072
	C vs. T				1.06(0.95–1.18)	Fixed	0.0	0.322
***PD-1*.*3* (rs11568821)**	AA vs. GG	4	1,280	1,236	1.72(0.50–5.94)	Random	59.4	0.394
Overall	AA vs. AG+GG				2.25(1.30–3.87)	Fixed	48.5	0.004
	AA+AG vs. GG				0.92(0.63–1.32)	Random	68.4	0.638
	AG vs. GG				0.79(0.65–0.96)	Fixed	0.0	0.021
	A vs. G				1.02(0.64–1.62)	Random	85.5	0.945

We also investigated the *PD-1*.*9* (rs2227982) and *PD-1* rs7421861 polymorphisms. It has been identified that *PD-1*.*9*, located in exon 5, is a non-synonymous SNP of *PD-1*, resulting the amino acid substitution from valine to alanine during protein synthesis, which probably lead to different structures and different functions of *PD-1*. As for *PD-1* rs7421861, it is situated in intron 1 where a number of regulatory elements and splicing control elements exist [45, 46]. Therefore, due to the disruption of the splice site or alteration of the mRNA secondary structure, *PD-1* rs7421861 may induce aberrant splicing, and further result in translational prevention [47–49]. However, we failed to find the associations between cancer risk and the *PD-1*.*9* (rs2227982) or *PD-1* rs7421861 in all genetic models and alleles. The limited sample size may be an important reason of the results, and we should treat the results with caution. Further studies are also needed to determine the function of these two polymorphisms.

In addition, we discussed the *PD-1*.*3* (rs11568821) polymorphism in our meta-analysis. The *PD-1*.*3* polymorphism was a guanine (G) to adenine (A) single nucleotide polymorphism (SNP) at nucleotide +7146 in the *PD-1* intron 4. A region of *PD-1* intron 4 was described as an enhancer-like structure containing binding sites for several transcription factors [50]. Existing study has shown that the *PD-1*.*3* polymorphism in this region may affect the binding of the runt-related transcription factor 1 (RUNX1) and alter the transcriptional regulation and the efficiency of *PD-1* gene [51]. Moreover, the research indicated that the presence of A allele of *PD-1*.*3* polymorphism disrupted the binding site for RUNX1 transcription factors and resulted the impairment of *PD-1* inhibitory effect and higher lymphocyte activity [50]. Hence, the A allele of *PD-1*.*3* polymorphism may have increased tumor immunity capacity and decreased the susceptibility of cancers. Consistently, our results of *PD-1*.*3* (rs11568821) polymorphism showed a decreased cancer risk in GA vs. GG, but an increased cancer risk was found in AA vs. AG+GG. Besides, no dramatic associations were found between AA vs. GG, AA+AG vs. GG genetic models or A vs. G allele and cancer risk. However, large scale and more rigorous analytical studies will be required to confirm the association between *PD-1*.*3* polymorphism and cancer risk.

There are some limitations should be addressed in this meta-analysis. First of all, the limited number of participants for *PD-1*.*3* (rs11568821) polymorphism may lead to insufficient statistical power to explore the real association. Secondly, the heterogeneities were significant in some genetic models and alleles for *PD-1*.*5* (rs2227981) and *PD-1*.*3* (rs11568821) polymorphisms. When we performed subgroup analyses stratified by ethnicity and control source, the heterogeneities in some subgroups were decreased or removed while in some subgroups were still existed. Thirdly, lacking of the original data limited our further evaluation of potential gene-gene, gene-environment, or even different polymorphism loci of the same gene, which all may affect cancer risk.

In summary, our meta-analysis suggested that the *PD-1*.*5* (rs2227981) polymorphism is associated with significantly decreased cancer risks both in TT vs. CC and TT vs. CT+CC genetic models, no matter for overall population, Asians subgroup or population-based subgroup, also the decreased cancer risk was found in T vs. C allele for overall population. No associations were found between the cancer risks and *PD-1*.*9* (rs2227982) or *PD-1* rs7421861 in all genetic models and allele. In addition, for *PD-1*.*3* (rs11568821) polymorphism, we found different cancer susceptibility between GA vs. GG and AA vs. AG+GG genetic models, and no associations between AA vs. GG, AA+AG vs. GG genetic models or A vs. G allele and cancer risk. However, our results firstly revealed a significantly decreased risk between *PD-1* polymorphisms and cancers, even though the data may be limited. Hence, large scale, well-designed epidemiological studies will be required to confirm our findings in the future.

## Supporting Information

S1 Fig Funnel plot for publication bias of the *PD-1*.*5* (rs2227981) polymorphism and cancer risk for overall populations (A for TT vs. CC; B for TT vs. CT+CC; C for TT+CT vs. CC; D for TC vs. CC and E for T vs. C). (TIF)

S2 Fig Funnel plot for publication bias of the *PD-1*.*5* (rs2227981) polymorphism and cancer risk for Asians subgroup (A for TT vs. CC; B for TT vs. CT+CC; C for TT+CT vs. CC; D for TC vs. CC and E for T vs. C). (TIF)

S3 Fig Funnel plot for publication bias of the *PD-1*.*5* (rs2227981) polymorphism and cancer risk for population-based subgroup (A for TT vs. CC; B for TT vs. CT+CC; C for TT+CT vs. CC; D for TC vs. CC and E for T vs. C). (TIF)

S4 Fig Funnel plot for publication bias of the *PD-1*.*9* (rs2227982) polymorphism and cancer risk for overall populations (A for TT vs. CC; B for TT vs. CT+CC; C for TT+CT vs. CC; D for TC vs. CC and E for T vs. C). (TIF)

S5 Fig Funnel plot for publication bias of the *PD-1* rs7421861 polymorphism and cancer risk for overall populations (A for CC vs. TT; B for CC vs. CT+TT; C for CC+CT vs. TT; D for CT vs. TT and E for C vs. T). (TIF)

S6 Fig Funnel plot for publication bias of the *PD-1*.*3*(rs11568821) polymorphism and cancer risk for overall populations (A for AA vs. GG; B for AA vs. AG+GG; C for AA+AG vs. GG; D for AG vs. GG and E for A vs. G). (TIF)

S1 Meta-Analysis on Genetic Association Studies Form (DOCX)

S1 Table Summary of the Egger's test *P*-value (DOCX)
