# Supplementary figures and images for: Programmed Cell Death-1 Polymorphisms Decrease the Cancer Risk: A Meta-Analysis Involving Twelve Case-Control Studies
Source: PLoS One. 2016 Mar 31;11(3):e0152448. doi: 10.1371/journal.pone.0152448 (PMC4816386; doi:10.1371/journal.pone.0152448)

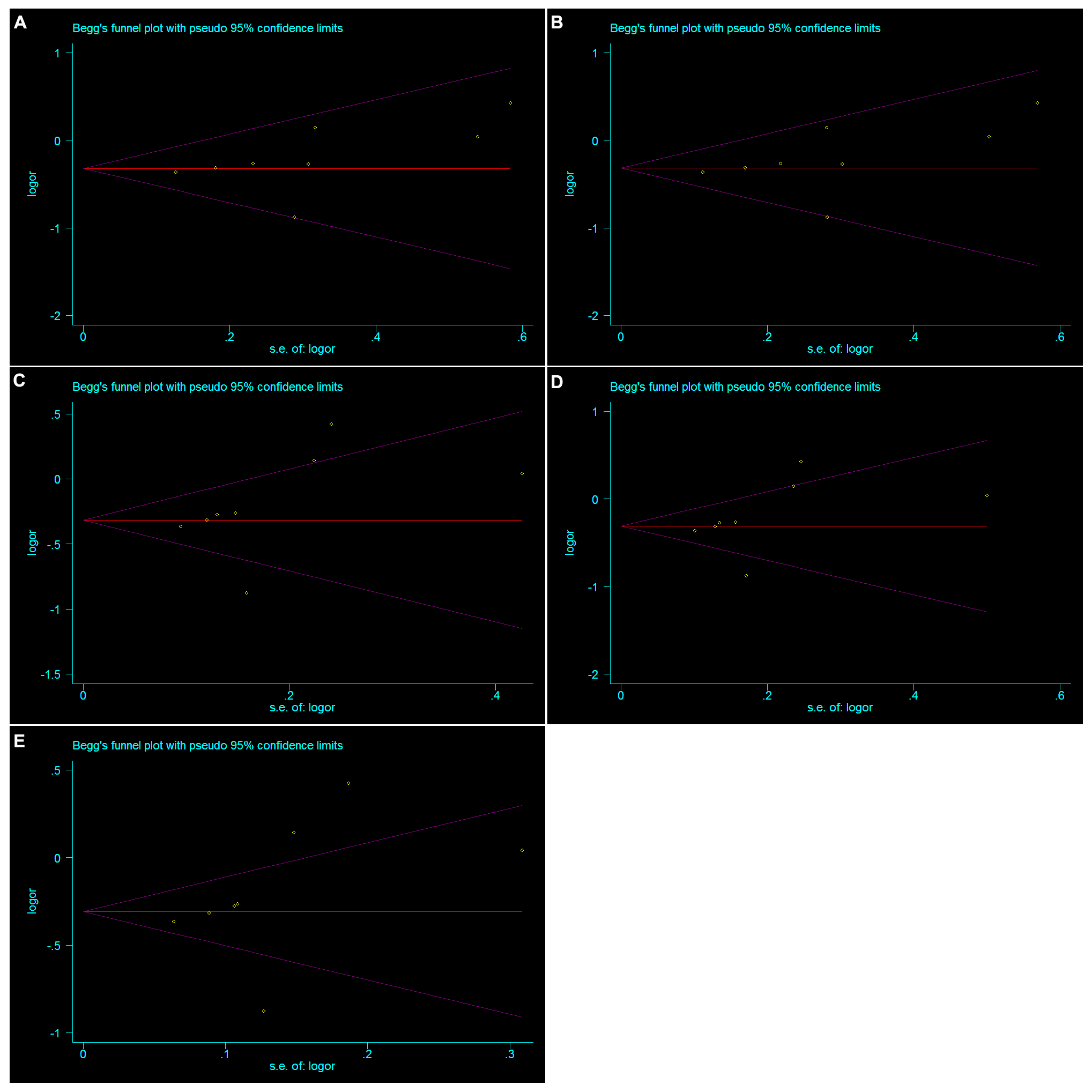

Supplement: S1 Fig — (TIF) [file pone.0152448.s001.tif]

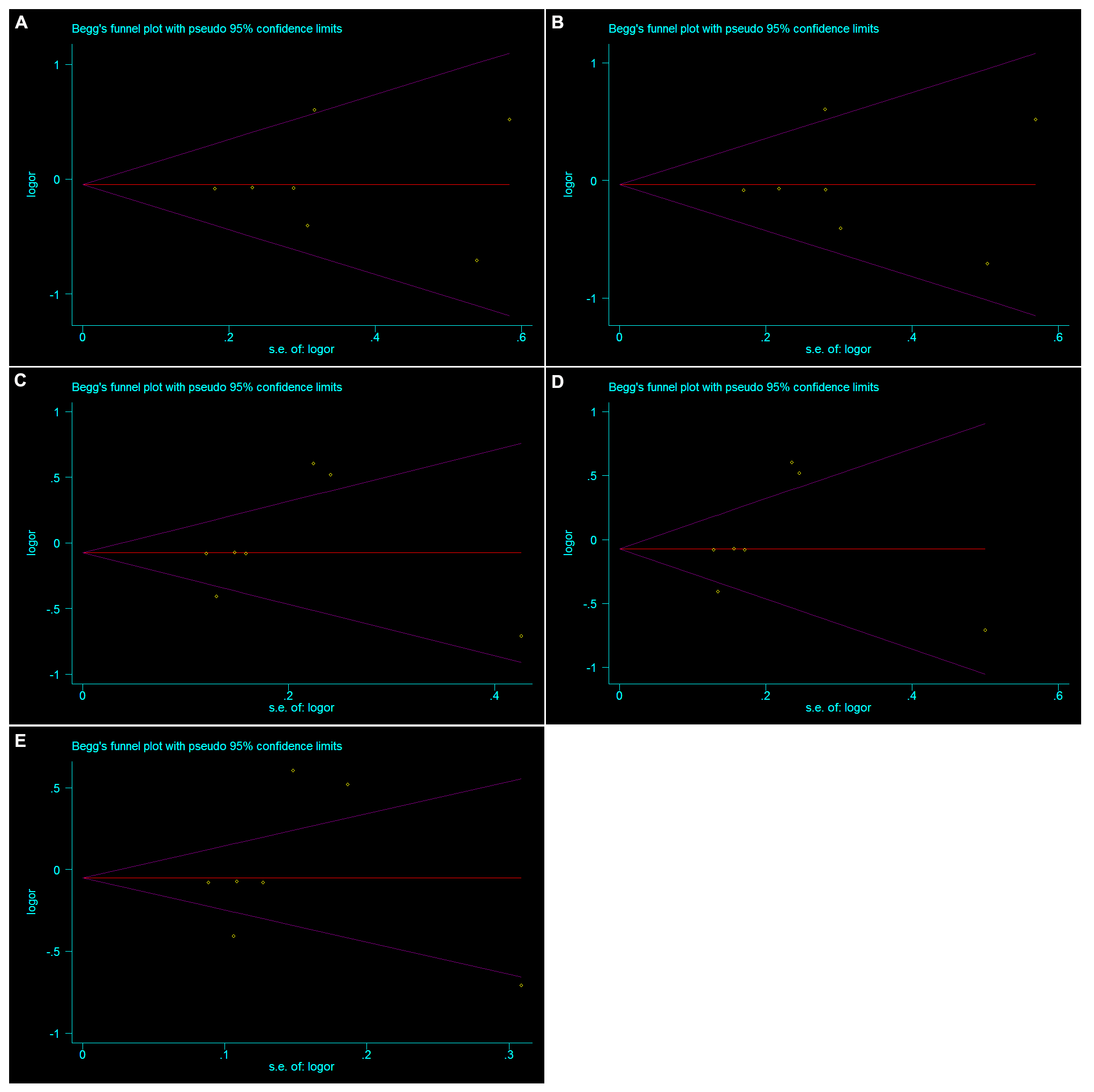

Supplement: S2 Fig — (TIF) [file pone.0152448.s002.tif]

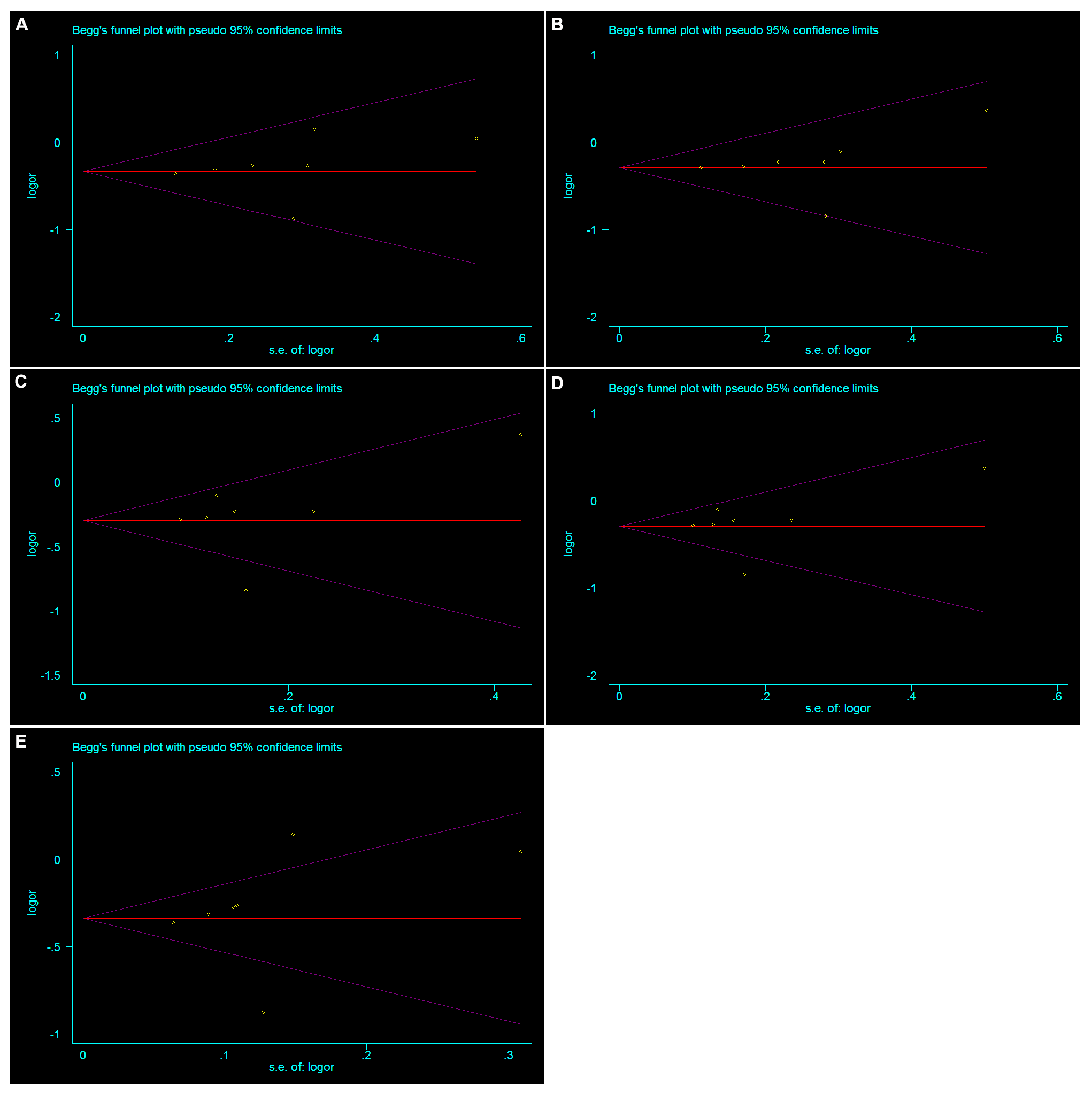

Supplement: S3 Fig — (TIF) [file pone.0152448.s003.tif]

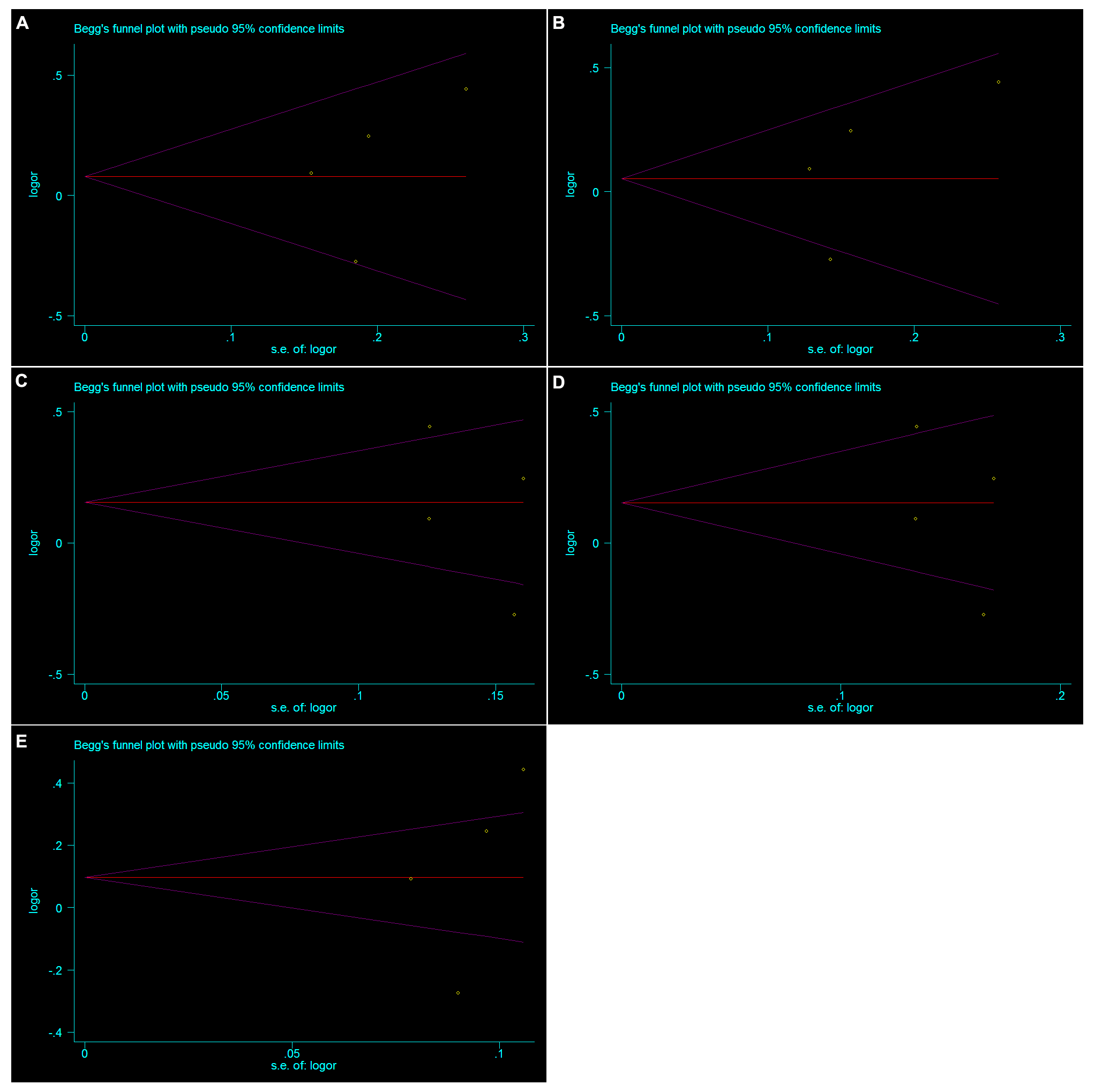

Supplement: S4 Fig — (TIF) [file pone.0152448.s004.tif]

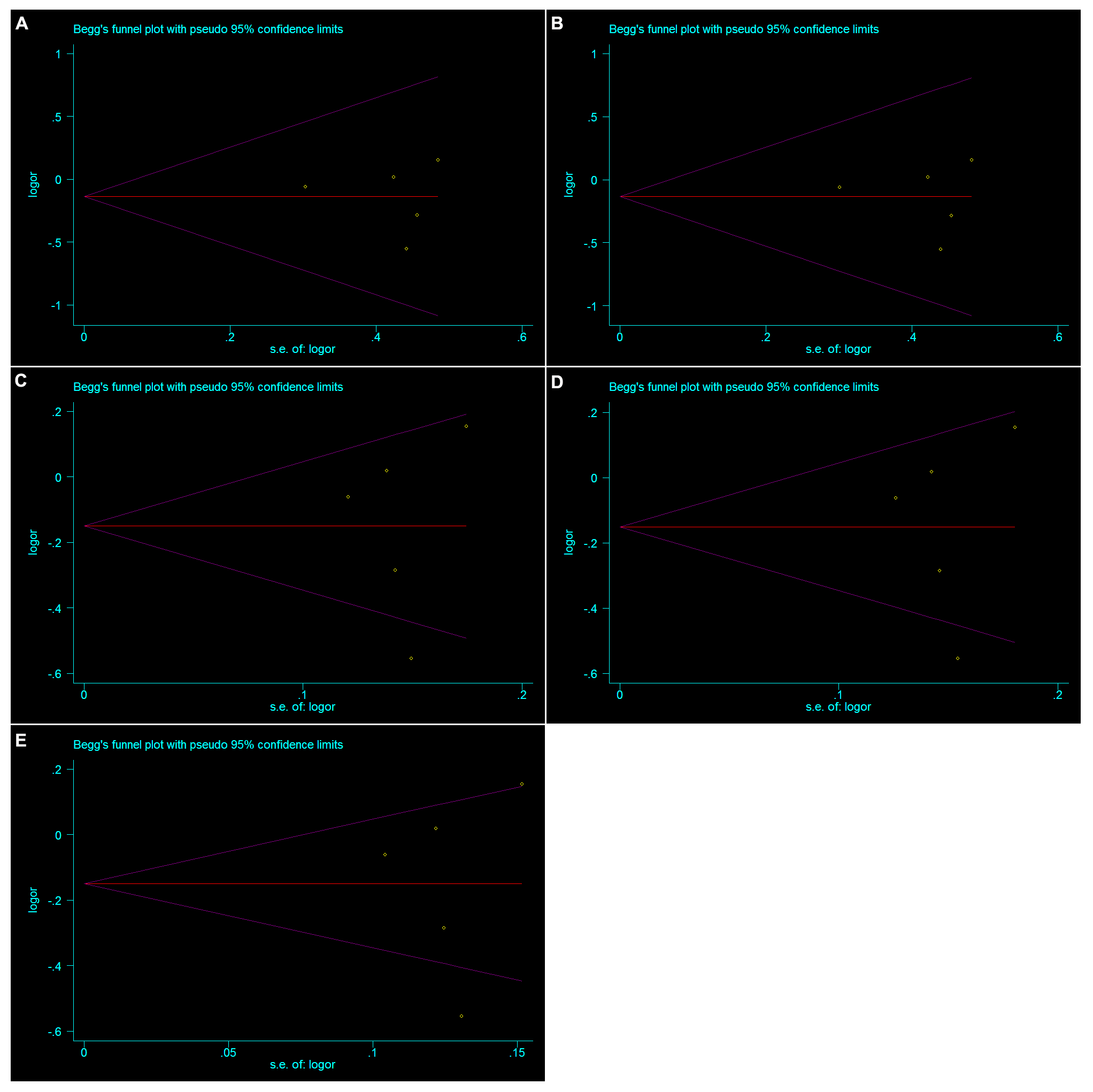

Supplement: S5 Fig — (TIF) [file pone.0152448.s005.tif]

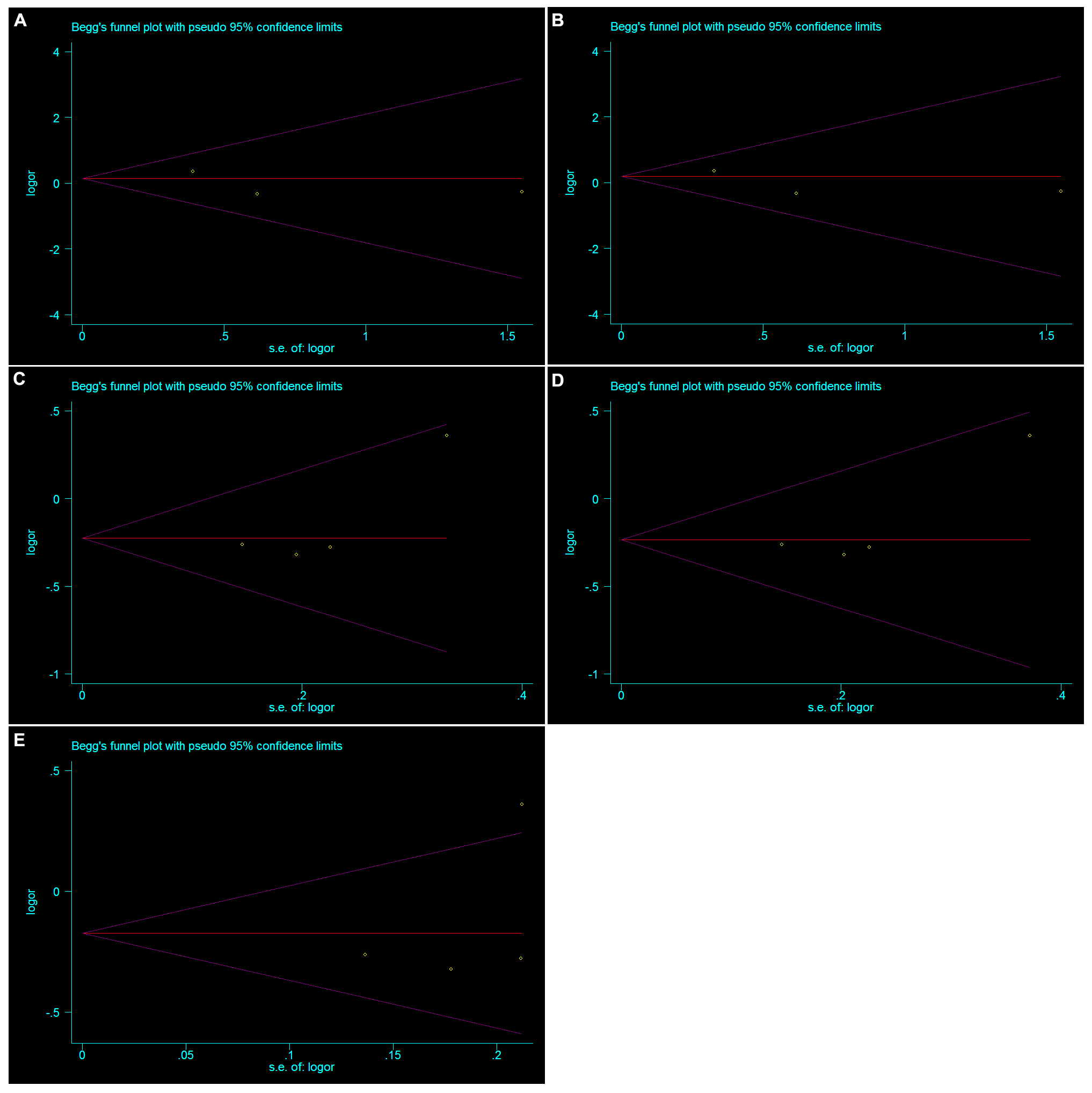

Supplement: S6 Fig — (TIF) [file pone.0152448.s006.tif]
