# Supplementary material for: Programmed Cell Death-1 Polymorphisms Decrease the Cancer Risk: A Meta-Analysis Involving Twelve Case-Control Studies
Source: PLoS One. 2016 Mar 31;11(3):e0152448. doi: 10.1371/journal.pone.0152448 (PMC4816386; doi:10.1371/journal.pone.0152448)
Supplement: S1 Table — (DOCX) [file pone.0152448.s008.docx]

**S1 Table Summary of the Egger's test *P*-value**

| **Group** | **Contrast** | ***P*-value of Egger's test** |
| --- | --- | --- |
| ***PD-1.5* (rs2227981)** |  |  |
| Overall | TT vs. CC | 0.296 |
|  | TT vs. CT+CC | 0.323 |
|  | TT+CT vs. CC | 0.252 |
|  | TC vs. CC | 0.285 |
|  | T vs. C | 0.286 |
|  |  |  |
| Asians Subgroup | TT vs. CC | 0.887 |
|  | TT vs. CT+CC | 0.975 |
|  | TT+CT vs. CC | 0.482 |
|  | TC vs. CC | 0.465 |
|  | T vs. C | 0.647 |
|  |  |  |
| Population-based Subgroup | TT vs. CC | 0.595 |
|  | TT vs. CT+CC | 0.702 |
|  | TT+CT vs. CC | 0.734 |
|  | TC vs. CC | 0.795 |
|  | T vs. C | 0.607 |
|  |  |  |
| ***PD-1.9* (rs2227982)** | TT vs. CC | 0.519 |
| Overall | TT vs. CT+CC | 0.453 |
|  | TT+CT vs. CC | 0.497 |
|  | TC vs. CC | 0.514 |
|  | T vs. C | 0.495 |
|  |  |  |
| ***PD-1* rs7421861** | CC vs. TT | 0.792 |
| Overall | CC vs. CT+TT | 0.79 |
|  | CC+CT vs. TT | 0.962 |
|  | CT vs. TT | 0.944 |
|  | C vs. T | 0.981 |
|  |  |  |
| ***PD-1.3* (rs11568821)** | AA vs. GG | 0.551 |
| Overall | AA vs. AG+GG | 0.484 |
|  | AA+AG vs. GG | 0.234 |
|  | AG vs. GG | 0.220 |
|  | A vs. G | 0.493 |
